# The ‘scientist’, the ‘analyst’ and the ‘novelist’: science or metrics?

**DOI:** 10.1007/s00125-022-05808-0

**Published:** 2022-12-22

**Authors:** Enzo Bonora

**Affiliations:** Division of Endocrinology, Diabetes and Metabolism, Department of Medicine, University of Verona and Hospital Trust of Verona, Verona, Italy

**Keywords:** Analyst, *h*-index, Meta-analysis, Metrics, Novelist, Review, Science, Scientist

## Abstract

An overwhelming number of meta-analyses and reviews are published by scientific journals. In part this may reflect some preference of editors and publishers for these types of papers, which are more frequently cited and can increase the impact factor of their journals. Meta-analyses and reviews are also attractive for investigators looking for a greater chance of having successful publications with several citations, and therefore an improved personal *h*-index. This greater ‘promise of success’ might have a deleterious effect on the intellectual maturation of investigators, particularly early career investigators, who might neglect original research and concentrate their efforts on meta-analyses and reviews. However, while meta-analyses and reviews are useful for emphasising data and disseminating concepts, progress in science requires original ideas, original experiments and original papers. ‘Analysts’ and ‘novelists’ are welcome, but ‘scientists’ are indispensable.

## Proliferation of scientific journals and papers

When I started my career in diabetes research in the late 1970s, only a dozen peer-reviewed scientific journals were devoted to the area of endocrinology, metabolism and diabetes. Some of these journals were not published monthly, and very often their issues contained no more than 10–12 papers. Additionally, there were only 30–35 peer-reviewed scientific journals worldwide in the area of general and internal medicine. Now, there are dozens of journals in the area of endocrinology, metabolism and diabetes and hundreds in the area of general and internal medicine [1]. Moreover, many of these journals publish the majority of their papers online, thus overcoming previous constraints on the number of accepted articles imposed by publishers when the cost of the production of paper journals had to be curbed. Remarkably, an increase in the number of published papers translates into increased profit for publishers as a result of page charges and other fees paid by authors or their institutions.

PubMed indexed 15,222 papers with the keyword ‘diabetes’ in the title published in the years 1980–1989 [2], and 124,718 published in the last 10 years (1 July 2012 to 30 June 2022) [3]. This is an increase of one order of magnitude. Several questions arise from this: is there a scientific justification for all these journals and all these papers—journals and papers which require thousands of qualified reviewers with specific expertise in more and more complex scientific areas. Are thousands of qualified reviewers really available? And if available, are they always willing to write impeccable reviews? And if the reviewing process is not impeccable, do such papers deserve publication?

It is reasonable to believe that the increased number of research products, papers and journals reflects a greater number of investigators in the field of diabetes, including in areas of the world where there have not been large amounts of such research in the past. Whilst an increase in the volume of research in specialist areas such as diabetes is obviously appreciated, an exponential increase in the number of research articles published may prove a challenge for the reader who wishes to remain up to date. In this respect, it is critical that a high quality of data is maintained in order to ensure scientifically relevant results are generated and to avoid the reader becoming overwhelmed by the volume of literature. This should be a priority for all referees and editorial boards.

The rate of progress in science and medicine is fast; many breakthroughs and seminal data become available every week. However, there is no doubt that many published papers are quite repetitive, if not redundant, and many do not provide a true step forward in our understanding. The tsunami of quite similar, if not conceptually identical, papers published on COVID-19 outcomes in diabetes is a good example [4]. The ‘nothing-to-add’ papers seem to be instrumental mainly for supporting some careers based upon personal metrics (number of publications and number of citations), and/or for the improvement of the metrics of some journals (mainly the impact factor). In this context, an interesting phenomenon surrounds meta-analyses and reviews, formats of articles which are increasingly populating even the most respected journals.

## Invasion of meta-analyses

In the past, meta-analyses were virtually non-existent. In the field of diabetes, no studies reporting a meta-analysis were available until the 1990s, with only 33 titles indexed in PubMed and published between 1990 and 1999 [5]. Today the contents list of most journals often includes one or more meta-analyses and many of them focus on the same topic. Of the 124,718 papers published in the field of diabetes in the last 10 years, 3890 (3%) were meta-analyses [6], with more than 100 on dipeptidyl peptidase-4 (DPP-4) inhibitors, glucagon-like peptide-1 (GLP-1) receptor agonists and/or sodium–glucose cotransporter 2 (SGLT-2) inhibitors [7–9]. It is quite often challenging to find substantial novelty when a meta-analysis is compared with previous ones on the same topic.

Meta-analyses are considered to be the best tool in evidence-based medicine and are therefore instrumental for guidelines and standards of care [10]. They often inspire statements issued by scientific societies, national and international medicine agencies and the WHO. Although they exist only because RCTs or observational studies have been published beforehand, providing the essential data, the efficiency of citing a meta-analysis, rather than searching for and assessing all the primary evidence, could potentially lead to the meta-analysis itself ranking more highly than the papers which reported the data it analysed [11]. As a consequence, authors of meta-analyses (the ‘analysts’) might receive more attention than authors of original papers (the ‘scientists’), despite the fact that meta-analyses are generally confirmatory. This is quite paradoxical. When meta-analyses are authored by people who did not contribute to the raw data analysis, or when they are authored by people who have never published original data in the field, the paradox might be met with some surprise, if not perplexity. In fact, it might sound like a type of exploitation, if not parasitism. ‘Analysts’ take advantage of the hard work of ‘scientists’ and overshadow them.

## Explosion of reviews

In the period 1980–1989, reviews represented 7% of all papers with the keyword ‘diabetes’ in the title retrieved from PubMed (1208 of 15,222) [12], a ratio of 1 to 14. In the last 10 years this ratio has decreased to 1 to 7 (19,314 reviews out of 124,718 papers) [13]. More and more reviews, both systematic and narrative, are published, but very often the topic is the same and, not uncommonly, the authors are also the same. A good example is the large number of reviews published on the metabolic syndrome and cardiovascular disease [14], or on non-alcoholic fatty liver disease (NAFLD) and cardiovascular disease [15]. In the past, distinguished investigators primarily published the results of their original research, and only occasionally wrote reviews. Moreover, after writing one review, they were generally satisfied and did not publish further reviews on the same topic for several years, or sometimes never again. Nowadays, some authors write multiple reviews on the same topic across a short period of time, and some reviews are written by people who have never acted as investigators in the field. Strictly speaking, these people (the ‘novelists’) quite often narrate the scientific achievements of others. It is evident that some of them inflate their CVs with a collection of reviews on the same topic, without presenting any substantially new concepts. This is somewhat intriguing because plagiarism (including self-plagiarism) is a form of research misconduct. However, some incorrigible ‘novelists’, using linguistic acrobatics, seek to escape detection by the software designed to identify plagiarism and successfully publish the same concepts on the same topic several times. Inverting paragraphs, changing words and rephrasing concepts seems to have become a new art form.

## Journals’ priorities

Meta-analyses and reviews generally receive many citations, and editors and publishers are very interested in citations because they increase the impact factor, and therefore the reputation, of their journals. There is competition among journals in their respective impact factor, and meta-analyses and reviews offer good opportunities to improve their standing. As a consequence, despite repetitions, redundancies and risk of plagiarism, many journals are delighted to accept reviews and meta-analyses and often appear to prefer them to original papers. ‘Scientists’, therefore, are sometimes neglected in favour of ‘analysts’ and ‘novelists’. The hard work of scientists is sometimes sacrificed on the altar of impact factor.

In some circumstances meta-analyses and reviews might be suspected to be promotional for a given product (medicine, device, etc.), and to have been carried out and written only to support an industry and its economic interests. Journals risk losing their reputation if readers perceive such behaviour. The issue of ties between authors (scientists, analysts, novelists), journals and industries, and their potential common, rather than conflicting, interests is receiving increased attention [16–18]. Disappointingly, belief in the existence of opaque ties and connivances between laboratories, authors, journals and industries is undermining the public credibility of science and scientists.

## A shortcut to ‘success’

Regretfully, the preference given to meta-analyses and reviews by journals might exert a deleterious role on the intellectual maturation of investigators, particularly early career investigators seeking to establish independence. Disappointingly, I see more and more people who believe that results and success should be achieved with the least possible effort and behave accordingly. Meta-analyses and reviews have no or minimal cost, are believed to have a greater probability of acceptance, to provide a greater number of citations and, therefore, to boost an individual’s *h*-index. During their career, investigators might be tempted to move their commitment from original research to meta-analyses and reviews with the goal of expanding their CVs and taking a shortcut to a better professional position (and to the ‘glory’). This cheaper and greater ‘promise of success’ is a disincentive for investigators to engage themselves in the true scientific arena. The arena where original ideas are born and blossom. The arena where scientists struggle with cells, animals and humans, using the tools of epidemiology and genetics, molecular biology and biochemistry, physiology and pathology, and clinical and experimental medicine. The arena where investigators try to address unanswered questions with their original methodologies, challenging what is unknown. The arena where researchers are proud to be the first in providing new data, proposing new hypotheses, identifying further unanswered questions and contributing to the progress of science.

## Proposals

Strategies to mitigate the invasion of meta-analyses and the explosion of reviews could include: (1) a cap on the number of these types of article in each issue of a journal; (2) an annual cap for each specific topic (e.g. not more than one or two meta-analyses and/or reviews per year on one topic); (3) a limit on the number of meta-analyses and reviews that contribute to the impact factor of a journal (e.g. only ten such articles per year); (4) the exclusion of citations of meta-analyses and reviews in the calculation of the journal impact factor, restricting all calculations solely to original papers; and (5) the exclusion of citations of meta-analyses and reviews from the calculations of the personal *h*-index. This would disincentivise the mass production of meta-analyses and reviews.

The proliferation of ‘nothing-to-add’ papers and repetitive meta-analyses/reviews might also be limited by a more careful and critical assessment of them by referees and editorial boards. A careful revision should be warranted even when it is requested by a journal that does not rank among the highest in the field. Quick, superficial or benevolent revisions should be avoided under all circumstances. Detailed reviews should be written by reviewers for all papers and all journals, without any personal calibration according to journal ranking. Redundancies might be eliminated by a more extensive and stringent application of artificial intelligence, automated text mining and machine learning by journals. Identifying and challenging plagiarism should be a commitment upheld by all journals. Additionally, for papers presenting original research, it could be an editorial requirement of all journals that authors include a section detailing the novelty of their data. The reader would then be able to identify immediately the paper’s contribution to the advancement of knowledge.

The scientific community might also wish to discuss whether it is reasonable for journals to accept meta-analyses or reviews in which none of the authors have generated any of the raw data and/or have never published in the specific scientific area. A limited personal scientific experience in the field and/or an excessive and uncritical enthusiasm for a methodology or a topic not sufficiently mastered might mean that the reader is exposed to mixed, if not distorted, messages.

Although attempts have been made to list predatory journals [19], and authors have the ability to check if a journal is a member of a recognised professional organisation committed to best publishing practice or if it is indexed in well-reputed electronic databases, scientific societies could perhaps provide guidance, if not a formal accreditation (a sort of blue stamp) to journals, in order to mitigate the proliferation of those of poor quality.

Additionally, mentors should provide stronger guidance on the selection of appropriate article types and the identification of predatory journals. The most experienced of us should prevent the newest investigators from developing an infatuation with the number of papers included in their CVs. Publishing dozens of fundamental papers should be encouraged, rather than collecting hundreds of less relevant papers. Originality, innovation and contribution to the progression of knowledge should be regarded more highly than metrics. The questions ‘How many papers have you published?’ and ‘What is your *h*-index?’ should be replaced by the questions ‘What original investigations have you carried out?’ and ‘What have you discovered?’.

Furthermore, investigators should be judged by the original research they publish in respected journals and not by meta-analyses and reviews. Coarse metrics should not prevail over a detailed assessment of the scientific achievements of investigators. The relevance of achievements, rather than metrics, should be predominant in the opinions expressed by grant- and award-assigning committees as well as university and hospital recruitment boards. The scientific community could propose and implement alternative metrics that might be better weighted towards original research that is novel and of high quality.

## Conclusion

When they are not a means to an end in themselves and instead provide additional knowledge, high quality meta-analyses and systematic reviews are certainly useful and welcome. In the presence of the current overflow of new data and the need to summarise and digest results, they are instrumental in the easy dissemination of concepts. However, I believe their numbers should be reduced and limited to those actually contributing to the clarification of doubts and/or to those that warrant new insights or create the premise for further original research. Replication of results is essential for the consolidation of knowledge, whereas replication of meta-analyses is not. Similarity in the presentation of concepts and ideas in reviews can be boring for the reader, and often does little to further understanding.

It should always be remembered that scientific progress is founded on original experiments and original papers. ‘Analysts’ and ‘novelists’ are fashionable and fascinating, but they could not exist without ‘scientists’.

Long live the brave, indomitable and romantic ‘scientist’!
